# Possible Involvement of a Mitochondrial Translation Initiation Factor 3 Variant Causing Decreased mRNA Levels in Parkinson's Disease

**DOI:** 10.4061/2010/491751

**Published:** 2010-06-14

**Authors:** Anna Anvret, Caroline Ran, Marie Westerlund, Ann-Christin Thelander, Olof Sydow, Charlotta Lind, Anna Håkansson, Hans Nissbrandt, Dagmar Galter, Andrea Carmine Belin

**Affiliations:** ^1^Department of Neuroscience, Karolinska Institutet, 171 77 Stockholm, Sweden; ^2^Neurology Section, Department of Clinical Neuroscience, Karolinska University Hospital, 171 76 Stockholm, Sweden; ^3^Department of Pharmacology, Sahlgrenska Academy, University of Gothenburg, 405 30 Gothenburg, Sweden

## Abstract

Genes important for mitochondrial function have been implicated in Parkinson's disease (PD). Mitochondrial translation initiation factor 3 (MTIF3) is a nuclear encoded protein required for the initiation of complex formation on mitochondrial ribosomes. Dysfunction of MTIF3 may impair mitochondrial function and dopamine neurons appear to be particularly vulnerable to oxidative stress, which may relate to their degeneration in PD. An association was recently reported between the synonymous rs7669(C>T) in MTIF3 and PD in a German case-control material. We investigated rs7669 in a Swedish Parkinson case-control material. The study revealed no significant association of the individual genotypes or alleles with PD. When comparing the combined TT/CT-genotypes versus the CC-genotype, we observed a significant association (*P* = .0473) with PD. We also demonstrated that the TT-genotype causes a significant decrease in MTIF3 mRNA expression compared to the CC-genotype (*P* = .0163). Our findings support the hypothesis that MTIF3 may be involved in the etiology of PD.

## 1. Introduction

Parkinson's disease (PD) is the second most common neurodegenerative disorder and affects 1-2% of the population over the age of 50 worldwide [1–4]. Several findings support mitochondrial involvement in PD. 

 For example, the metabolite of the neurotoxin 1-methyl-4-phenyl-1,2,3,6-tetrehydropyridine (MPTP); 1-methyl-4-phenylpyridinium (MPP^+^) is a mitochondrial respiratory chain complex I inhibitor and causes degeneration of dopamine (DA) neurons in substantia nigra (SN) [5]. Another example is the pesticide rotenone, which also is a complex I inhibitor, causing nigrostriatal degeneration in rodents resembling the pathology of PD [6]. Mitochondrial DNA (mtDNA) encodes two ribosomal RNAs (rRNAs), 22 transfer RNAs (tRNAs) and 13 proteins. The mtDNA encoded proteins are all part of the oxidative phosphorylation system responsible for generating energy by aerobic metabolism, whereas the rRNAs and tRNAs are needed for intramitochondrial protein synthesis [7]. Activity of complex I in the electron transport chain has been reported to be reduced in SN and in platelets from patients with PD [8]. Certain mtDNA polymorphisms have been reported to be associated with PD, others to reduce the risk [9]. Further evidence to support mitochondrial dysfunction in PD comes from reports associating PD with genetic variants in DNA polymerase gamma protein (POLG), which is important for mtDNA replication and repair [10]. The N-terminal of the human POLG protein contains a short polyglutamine domain encoded by a CAG-repeat. It has been reported that rare length variants of the CAG-repeat in the POLG gene are associated with idiopathic PD [10]. Conditional knockout mice with a disruption of the mitochondrial transcription factor A (Tfam) in DA neurons (“MitoPark” mice) lend additional support to the hypothesis of mitochondrial involvement in PD [11]. These mice have respiratory chain deficiency in midbrain DA neurons and show Parkinson-like motor disabilities, which are relieved by L-dopa [11]. 

MTIF3 is required for the initiation complex formation on 55S mitochondrial ribosomes [12]. Dysfunction of MTIF3 may affect the expression of mtDNA-encoded proteins, which in turn may cause oxidative stress. Dopamine neurons appear particularly vulnerable to oxidative stress, which may be the cause of their degeneration in PD. To date, five nuclear genes identified at PARK loci have been suggested to directly or indirectly influence mitochondrial function: *α*-synuclein, Parkin, DJ-1, PINK1, and LRRK2 [13–15]. Mutations in the serine-threonine kinase PINK1 gene have been reported to be responsible for PARK6-associated autosomal recessive PD. The PINK1 protein is localized to the mitochondria in mammalian cells [16]. The serine-threonine kinase domain of PINK1 and the ortholog protein in *drosophila melanogaster *CG4523 have a 300 amino acids long homology region. This PINK1 homologue interacts with CG11656-PA, whose closest mammalian ortholog protein is MTIF3, supporting the hypothesis that MTIF3 is an interactor protein for PINK1 and important in the etiology of PD [17]. The aim of the present study was to analyze the MTIF3 synonymous polymorphism rs7669 (Asp266Asp, C > T) in a Swedish PD case-control material based on the reported association with sporadic and familial PD in a German case-control material [17]. In addition, we also investigated possible differences in mRNA expression levels between the three genotypes using cell lines from PD patients and controls.

## 2. Material and Methods

### 2.1. DNA Material

The polymorphism rs7669 (C > T) in MTIF3 was investigated in a Swedish PD case-control material. The ethical committees of each institution, Karolinska Institutet Forskningsetikkommitté Nord and Forskningsetikkommittén, University of Gothenburg, approved the study and each subject signed an informed consent. All subjects were unrelated and of Swedish origin. A total of 381 PD patients were recruited: 211 at Karolinska University Hospital, Stockholm and 170 at Sahlgrenska Hospital, Gothenburg (mean age at sample collection, 67.7 years; mean age of onset 59.3 years; 59.3% men). All sporadic PD patients fulfilled the “Brain Bank Clinical Diagnostic Criteria” for idiopathic PD [18]. Control subjects were blood donors, spouses to PD patients or individuals visiting hospitals or care centers for non-neurological symptoms in Stockholm (*n* = 135) and Gothenburg (*n* = 187) (*n* = 322; mean age at sample collection, 58.7 years; 43.8% men). 52 of the Stockholm PD patients and 38 of the Gothenburg PD patients had a self-reported familial history of PD with one or more first-, second- or third-degree relatives with PD. DNA was extracted from blood samples according to standard protocol.

### 2.2. Pyrosequencing

To genotype the genetic variant rs7669 in MTIF3 we used pyrosequencing, a method that analyzes genetic variants by detecting the energy released when a nucleotide is incorporated into a predefined DNA strand [19]. To test for genotyping errors we used water as negative controls and resequenced some of the samples chosen at random to confirm the results. The following primer sequences have been used; forward primer 5′-CGTGCTTTCAGCAAAAATG -3′, biotinylated at the 5′-end; reverse primer 5′- AAAGGACTGCAGACCAAGGA -3′, and sequencing primer 5′- TCCTTATCATTTCCA -3′. Polymerase chain reaction (PCR) was carried out with Taq polymerase after which the biotinylated PCR product was immobilized on streptavidin-coated beads according to manufacturer's instructions. The immobilized DNA template was captured onto filter probes (PyroMark Vacuum Prep Tool, Biotage AB, Uppsala, Sweden). The filter probes were flushed with 70% ethanol, denaturation solution, washing buffer, and the single-stranded template was annealed to a reverse sequencing primer. All solutions were prepared according to manufacturer's instructions (Biotage AB, Uppsala, Sweden). Samples were analyzed on an automated pyrosequencer using a PSQ 96 System together with SNP Software and SNP Reagent Kits (Biotage AB, Uppsala, Sweden). Genotype distribution and allele frequencies were compared between the different groups using the Chi-square (*χ* ^2^) test [20]. Distribution of genotypes in controls was tested for consistency with the Hardy-Weinberg equilibrium. Statistical significance levels were set at *P* < .05.

### 2.3. Prediction of mRNA Secondary Structure

To evaluate the effect of rs7669 on the mRNA structure, the secondary structure of MTIF3 was predicted using the publicly available online mfold program (version 3.2) [21, 22]. Partial MTIF3 mRNA sequences of 141 nucleotides including flanking sequences (70 nucleotides) on either side of the polymorphism were analyzed and compared to wild-type sequence.

### 2.4. Epstein-Barr Virus Transfection and Culture of Human B-lymphocytes

B-lymphocytes were separated from peripheral blood by standard protocols using Ficoll-Paque (GE Healthcare Bio-Sciences Corp., Piscataway, NJ, USA). The cells were cultivated in RPMI 1640 medium (SIGMA, St. Louis, MO, USA) with 20% fetal calf serum and L-glutamine (200 mM; Invitrogen, Carlsbad, CA, USA), penicillin—streptomycin (5000 *μ*g/ml; Invitrogen, Carlsbad, CA, USA), cyclosporine (1 *μ*l/ml; Apoteket, Stockholm, Sweden), and filtered supernatant of Epstein-Barr virus (EBV) infected B95-8 cells. The medium was changed twice a week until cell lines were established at which time the cells were gradually frozen and kept at −140°C until use. For mRNA quantification, the cells were thawed, cultured for approximately two weeks, and harvested when cell number reached 4-5 million cells.

### 2.5. Quantitative Real-Time PCR

Quantitative real-time PCR (qRT-PCR) was performed on RNA from EBV transfected B-lymphocytes from individuals representing the three different rs7669 genotypes (CC, CT, and TT). Quantification of MTIF3 mRNA expression levels for the three different genotypes was performed in samples from six different individuals per genotype. Individuals were chosen only based on their rs7669 genotype regardless their neurological status (CC genotype: 2 PD patients and 4 controls, CT genotype: 4 PD patients and 2 controls, and TT genotype: 5 PD patients and 1 control). Total RNA was isolated from the EBV-transfected B-lymphocytes using RNeasy Mini Kit (Qiagen, Hilden, Germany) and quantified by spectrophotometry at 260 nm. cDNA was generated from 1 *μ*g RNA by a modification of the manufacturer's protocol using Deoxyribonuclease I, Amplification Grade (Invitrogen, Carlsbad, CA, USA), and SuperScript III Platinum Two-Step qRT-PCR kit with SYBR Green (Invitrogen, Carlsbad, CA, USA). qRT-PCR was performed on an ABI Prism 7000 (Applied Biosystems, Foster City, CA, USA) using SYBR Green I dye (Invitrogen, Carlsbad, CA, USA). The samples were run in triplicates for the target gene MTIF3 (forward primer 5′- ATCGCTTGCCCCAGCAC -3′; reverse primer 5′- TCATCCCCAGTTGATGAGG -3′) and two housekeeping genes; beta-actin (forward primer 5′- AACCGCGAGAAATCATGTTTG -3′; reverse primer 5′- CAGAGGCGTACAGGGATAGCA -3′) and cyclophilin (forward primer 5′- GACCCAACACAAATGGTTCC -3′; reverse primer 5′- GGCCTCCACAATATTCATGC -3′). Amplification of a single gene product was confirmed by monitoring the dissociation curve as well as agarose gel electrophoresis. Threshold cycle (Ct) values from the exponential phase of the PCR amplification plot were analyzed with ABI Prism 7000 SDS v1.2.3. Each Ct value for the target transcripts was normalized to beta-actin and cyclophilin, using qBase v1.3.5 [23], based on the 2^−ΔΔCt^ method [24]. The 2^−ΔΔCt^ method has been extended in the qBase software to include multiple stable expressed reference genes to improve normalization [23]. The quantification of the expression was compared between the three different genotypes using Student's *t*-test. Significance level was set at *P* < .05.

## 3. Results

### 3.1. Genotyping and Secondary Structure of mRNA

We found all three genotypes of rs7669 (CC, CT and TT) in both PD cases and controls. The observed frequencies of the controls were in agreement with the Hardy-Weinberg equilibrium (data not shown). Our analysis showed no significant association of any of the three individual genotypes or alleles with PD (Table 1). When comparing the TT/CT genotypes versus the CC genotype, we observed a significant association with sporadic PD (*P* = .0473). Stratification by PD onset into 50 years or younger versus older than 50 years of age at onset compared to age-matched controls revealed no associations of genotype or allele distribution with disease (data available upon request). 

To test whether the synonymous cytosine798thymine (Asp266Asp) substitution of rs7669 change the secondary structure of mRNA we did an mfold analysis of MTIF3 mRNA. Our analysis indicates that rs7669 in exon 5 results in an apparent change in the mRNA secondary structure and slightly higher energy for mRNA folding (dG) compared with the dG of the wild-type mRNA folding (data available upon request).

### 3.2. RNA Expression

**Figure 1:**
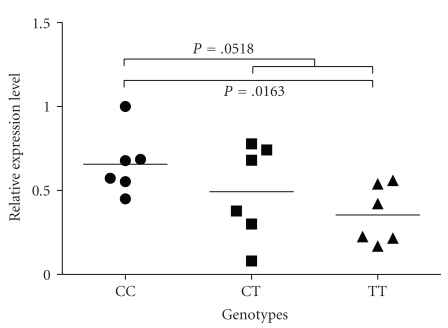
Quantification of MTIF3 mRNA levels. MTIF3 mRNA levels in Epstein-Barr virus (EBV) transfected B-lymphocytes from individuals carrying the three different rs7669 genotypes, normalized to the housekeeping genes beta-actin and cyclophilin. mRNA levels were found to be significantly lower in TT genotypes compared to CC genotypes (*P* = .0163) using Student's *t*-test.

To further investigate possible consequences of the synonymous polymorphism rs7669 we studied transcriptional activity of the MTIF3 gene by measuring mRNA expression levels using qRT-PCR. We found that MTIF3 mRNA levels were significantly lower (*P* = .0163) in cells from individuals carrying the TT genotype compared to individuals with the CC genotype (Figure 1). No significant difference of MTIF3 mRNA levels was found between cells from individuals carrying CT and CC genotypes (*P* = .266). Testing both T-containing genotypes together (TT/CT) versus CC, the MTIF3 mRNA decrease was close to significance (*P* = .0518). Similar results were observed using the Mann-Whitney *U* test. Since individuals were chosen based on their rs7669 genotype without restriction to their neurological status, we also compared expression data from patients versus controls, which showed no significant association (*P* = .0795).

## 4. Discussion

Our findings strengthen the hypothesis that the MTIF3 rs7669 variant may be involved in the etiology of sporadic PD, because of the significant association of the TT/CT versus the CC genotype in a Swedish sample set. Abahuni et al. (2007) have previously investigated the MTIF3 rs7669 polymorphism in a German case-control material consisting of patients with positive familial history of PD as well as sporadic cases [17]. The previous study found no significant association of the TT/CT genotypes with sporadic PD (*P* = .0540), while results from our data set showed a significant association (*P* = .0473). Interestingly, Abahuni et al. (2007) showed an association of increased CT genotypes and decreased TT genotypes among sporadic cases (*P* = .0345) and in combined samples (*P* = .0073) compared to controls. 

The rs7669 variant might be in linkage disequilibrium with some other real disease causing locus in MTIF3 or in some other gene in its vicinity and this locus may differ between ethnical populations, although no other known MTIF3 polymorphisms have been reported to be in linkage disequilibrium with rs7669 on chr13: 26,907,780–26,922,711. The results from the two studies are contrary and could possibly be explained by that the two sample series were collected from different countries and there might be a small effect of background population on the association, although this is unlikely as both our study and the previous one report similar allele frequencies that are in Hardy-Weinburg equilibrium. 

To date, the only two genetic studies on MTIF3 and PD are Abahuni et al. (2007) and the present study. Our results indicate that the TT/CT genotypes might be more common among sporadic PD cases than controls, strengthening the involvement of mitochondrial dysfunction in PD. We could also show that the B-lymphocytes from individuals carrying the TT genotype contain significantly less MTIF3 mRNA compared to cells from individuals carrying the CC genotype (*P* = .0163). This suggests that the TT genotype affects MTIF3 mRNA levels although it cannot be fully excluded that individuals with this genotype also carry additional genetic variants affecting the mRNA levels. By dividing the samples into patients and controls without respect to their genotype, we show that the decrease in expression of MTIF3 mRNA is not due too PD (*P* = .0795). Mechanisms by which synonymous polymorphisms predispose to disease are not well understood, but several possibilities have been suggested including that they might affect mRNA transcriptional activity, mRNA stability, secondary mRNA structure, or at the protein level, synthesis, folding, and thereby turnover or function [25]. We performed a structure analysis *in silico* to investigate if the synonymous polymorphism rs7669 in MTIF3 could induce an alteration of the secondary mRNA structure. Our findings indicate that rs7669 results in a change the in mRNA secondary structure and that the polymorphism has the potential to affect folding and mRNA stability. However, no strong conclusions can be drawn from the artificial conditions used for modeling secondary structures *in silico*.

## 5. Conclusion

In summary we show that a genetic variant of MTIF3 is associated with sporadic PD in Sweden, supporting mitochondrial involvement in the disease. We have also demonstrated that the TT genotype leads to a decreased expression of MTIF3 mRNA. Further genetic studies are needed in larger materials to confirm these genetic findings. Studies of the MTIF3 protein at the cellular level are also needed to understand possible cellular sites at which alterations of MTIF3 levels may affect function.

## Figures and Tables

**Table 1:** Association analysis of MTIF3 rs7669 genotype and allele frequencies in Parkinson's disease (PD).

MTIF3	Familial PD (*n*= 90)	Sporadic PD (*n*= 291)	Combined samples (*n*= 381)	Controls (*n*= 322)
Genotypes				
CC (%)	59 (65.6)	168 (57.7)	227 (59.6)	211 (65.5)
CT (%)	28 (31.1)	108 (37.1)	136 (35.7)	96 (29.8)
TT (%)	3 (3.3)	15 (5.2)	18 (4.7)	15 (4.7)
*χ* ^2a^	*P* = .8513	*P* = .1335	*P* = .2439	
Alleles				
C (%)	146 (81.1)	444 (76.3)	590 (77.4)	518 (80.4)
T (%)	34 (18.9)	138 (23.7)	172 (22.6)	126 (19.6)
*χ* ^2b^	*P* = .8393	*P* = .0778	*P* = .1693	
Odds ratio	1.045 (0.686–1.59)	0.783 (0.596–1.03)	0.834 (0.644–1.08)	
Combined genotypes				
CC/CT (%)	87 (96.7)	276 (94.8)	363 (95.3)	307 (95.3)
TT (%)	3 (3.3)	15 (5.2)	18 (4.7)	15 (4.7)
*χ* ^2b^	*P* = .5867	*P* = .7761	*P* = .9853	
Odds ratio	1.42 (0.401–5.01)	0.899 (0.432–1.87)	0.985 (0.488–1.99)	
TT/CT (%)	31 (34.4)	123 (42.3)	154 (40.4)	111 (34.5)
CC (%)	59 (65.6)	168 (57.7)	227 (59.6)	211 (65.5)
*χ* ^2b^	*P* = .9961	*P* = .0473	*P* = .1049	
Odds ratio	0.999 (0.612–1.63)	1.39 (1.01–1.93)	1.29 (0.948–1.75)	

^a^Chi-square test (*χ* ^2^) (2DF) for 2 × 3 contingency table, ^b^ *χ* ^2^ (1DF) for 2 × 2 contingency table.
